# Efficacy of transdiagnostic cognitive-behavioral therapy for assertiveness: A randomized controlled trial

**DOI:** 10.1016/j.invent.2023.100629

**Published:** 2023-05-13

**Authors:** Tobias Hagberg, Patrik Manhem, Martin Oscarsson, Fiona Michel, Gerhard Andersson, Per Carlbring

**Affiliations:** a Department of Psychology, Stockholm University, Sweden; d Centre for Clinical Interventions, Australia; c Department of Clinical Neuroscience, Karolinska Institute, Stockholm, Sweden; d Department of Behavioral Sciences and Learning, Department of Biomedical and Clinical Sciences, Linköping University, Linköping, Sweden

**Keywords:** Assertiveness, Assertive behavior, Anxiety, Depression, Stress, Avoidance

## Abstract

Assertiveness training has been an essential component in cognitive-behavioral therapy (CBT), for example, in the treatment of social anxiety and in dialectical behavioral therapy. However, the assertiveness construct has garnered little attention in recent clinical research. The objective of this study was to investigate the efficacy of an eight-week transdiagnostic stand-alone internet-based CBT intervention specifically aimed at increasing levels of assertive behavior. Following inclusion, we randomized *N* = 210 participants into three groups: therapist-guided self-help, unguided self-help, and a wait-list control condition. After a one-year follow-up, we employed a linear mixed model to estimate the effects at both post-test and follow-up for the primary outcome measures of assertiveness, Adaptive and Aggressive Assertiveness Scales, the Rathus Assertiveness Schedule, and secondary outcome measures of anxiety, depression, and general well-being. We also assessed reliable clinical change. Compared to the wait list at the post-treatment, estimated between-group effect sizes on self-rated adaptive assertiveness were statistically equivalent for the two treatment groups both at the post and at the one-year follow-up time points, ranging from ES = 0.95 to 1.73, with reliable clinical recovery proportions from 19 % to 36 %. The increase in aggressive assertiveness ranged from ES = 0.62 to 0.90 compared to the wait-list condition at post. For social anxiety symptoms, the effects compared to the wait list at post-treatment ranged from ES = 0.67 to 0.93, with a reliable clinical recovery rate from 16 % to 26 %. For self-assessed well-being, the effects compared to the wait list at post ranged from ES = 0.70 to 1.05. No effects were observed for generalized anxiety, although within-group evidence was found for a medium effect on depression one year after treatment. Overall, the two treatment conditions produced similar effects. In general, participation increased healthy assertive expressions regardless of treatment condition, all the while reducing self-assessed social anxiety and, over time, possibly also depression. Participation also improved general well-being. The findings demonstrate that the assertiveness construct can be a suitable target for intervention, with reductions of both psychiatric symptoms and non-syndromal problems in daily life. The study was preregistered at ClinicalTrials.gov (NCT04240249).

## Highlights

• Standalone internet-based cognitive-behavioral therapy (iCBT) effectively increases adaptive assertiveness levels
• iCBT assertiveness training notably curbs social anxiety, aiding clinical recovery for many.
• Positive effects persist at one-year follow-up
• Assertiveness as a construct merits renewed clinical research attention
• Assertiveness could be a valuable target in a transdiagnostic self-help guide without requiring therapist support.

## 1 Introduction

Escape and avoidance is associated with the development of maladaptive behavior among vulnerable participants in laboratory trials (Sheynin et al., 2014), as well as psychopathologies in clinical presentations. Experiences of stress, anxiety, and depression are often associated with avoidance of constructively presenting one's thoughts, feelings, needs, and wishes in relation to others, that is deficits in assertive behavior (Speed et al., 2018). Assertiveness can be difficult to delineate from social skills in general (Linehan, 1979), but a common definition is “direct, firm, positive […] action [enabling] us to act in our own best interests, to stand up for ourselves without undue anxiety, to exercise personal rights without denying the rights of others, and to express our feelings and needs […] honestly and comfortably” (Alberti and Emmons, 2017, p. 34). Examples include politely saying “no” to a boss requesting undue overtime, actively participating in social activities, accepting/acknowledging a compliment without deflecting, and verbalizing feelings in personal relationships without acting out. A lack of assertiveness is associated with several psychological problems, including stress, generalized anxiety, social anxiety, depression, and panic disorder, as well as emotional instability, strained relationships, and low self-esteem (Speed et al., 2018). While there are diagnoses, diagnostic tools, and treatment manuals for conditions associated with these problems, no evidence-based interventions specifically target assertiveness for a broader population.

Assertiveness training dates back to the very first behavioral therapies (e.g., Salter, 1949; Wolpe and Lazarus, 1966). In the 1970s, the concept was popularized in self-help books (e.g., Alberti and Emmons, 1974; Fensterheim and Baer, 1975; Smith, 1975). Research on assertiveness training peaked in the 1980s (Speed et al., 2018). Although the behavioral techniques of the first wave of therapy were supplemented by cognitive restructuring techniques (e.g., Beck, 1979), in the following decades, techniques such as modeling and behavior rehearsal remained active parts of treatments for psychological syndromes such as anxiety disorders and depression. Linehan's (1979) manual for assertion therapy combining behavior rehearsal with cognitive restructuring was a stepping stone toward Dialectical Behavior Therapy (DBT), in which assertion skills training in a group setting is integral (Linehan, 1993).

Assertion is regarded as a situation-specific trait rather than a generalized trait (Hull and Hull, 1978). Building on the definition by Alberti and Emmons (2017), assertiveness can be operationalized as acting with respect to personal rights without infringing on the rights of others. Constructive assertion takes into account both the desired result of the interaction (e.g., saying “no” to someone else's demand or making a request) and the intensity of the interaction, where the latter is calibrated with regard to both the importance of the relationship and what Linehan (1993) refers to as self-respect. Using this definition, assertive behavior can be thought of as the product of respect for the rights of others and respect for one's own rights. This definition offers several opportunities for idiographic and contextual descriptions of assertion in therapy (i.e., when designing in vivo behavioral experiments, regardless of cultural influences on what is considered acceptable behavior within a family, community, society, etc.) (Mitamura, 2018; Sigler et al., 2008).

Speed et al. (2018) concluded that while assertiveness training is part of DBT, as well as acceptance and commitment therapy (ACT), general assertiveness training is not often mentioned in the current CBT literature. Few studies on assertiveness training have been published since the early 1980s. Recent exceptions include Baker and Jeske (2015), showing a negative relationship between social anxiety and assertiveness. Vagos and Pereira (2019) reported a negative association between mental distress in general and assertiveness, and Antúnez (2020) found a link between circadian typology and level of assertiveness. Speed et al. (2018) further concluded that there is potential for assertiveness training as an intervention for individuals suffering from anxiety and depression, and as a means to increase relationship satisfaction. The lack of contemporary evidence for the assertiveness construct and assertiveness training as a transdiagnostic intervention calls for new research on the subject.

While the empirical support for assertiveness training is scarce at best, there is much evidence for the effectiveness of CBT for symptoms and syndromes associated with inadequate assertiveness. In a review of meta-analyses, Hofmann et al. (2012) conclude that CBT is one of the most effective forms of therapy. This includes its application for symptoms related to trauma and stress, as well as syndromes related to depression and anxiety. A review by Andrews et al. (2018) also lends support to internet-delivered CBT (iCBT) for anxiety and depression, showing an average between-group effect size of g = 0.80 compared to the controls. Carlbring et al. (2018) also show that iCBT, on average, produces equivalent overall effects compared to face-to-face treatment. iCBT has proven effective in both guided and unguided applications (i.e., with or without therapist support), although guided iCBT tends to produce slightly larger effects (Baumeister et al., 2014). iCBT has also proven effective in transdiagnostic applications, including interventions targeting stress (Day et al., 2013), procrastination (Rozental et al., 2015), and perfectionism (Rozental et al., 2017). The Western Australian Centre for Clinical Interventions offers various self-help resources for mental health problems. These resources include Assert Yourself (Michel and Fursland, 2008), a series of 10 modules with concepts and strategies primarily based on cognitive behavioral therapy (CBT), with a focus on assertiveness.

This study aimed to conduct a randomized controlled trial (RCT) on the effects of an eight-week iCBT intervention targeting unhealthy assertiveness, Respekt^2^ (Respect Squared), based on the Michel and Fursland (2008) modules. We tested the effects on measures of anxiety, depression, and well-being, as well as the difference between guided and unguided iCBT against the control group (three-armed trial).

## 2 Method

### 2.1 Design

We randomly allocated participants to three groups: (1) guided self-help, (2) unguided self-help, and (3) eight-week wait-list control. The final sample consisted of 210 participants, with 70 participants per group. The group sizes were based on an a priori power calculation according to guidelines for linear models outlined in Cohen (1988), assuming a between-group effect size of Cohen's d of 0.80 on the Adaptive and Aggressive Assertiveness Scales (AAA-S; Thompson and Berenbaum, 2011), power .90, alpha .05, and a 15% “worst case” drop-out rate per week, with the duration of the intervention being eight weeks in total.

The study was registered at ClinicalTrials.gov (NCT04240249). An error in the design was corrected post-registration, in that the Rathus Assertiveness Schedule (RAS) scale was moved from the secondary outcome measures category to the primary outcome measures category, as the scale captures the primary dependent variable of interest. Before recruitment started, the study received ethical approval from the Swedish Ethical Review Authority (Diary number: 2019-05165).

### 2.2 Participants

Participants were recruited from the public through advertisements on social media and other websites. Interested individuals were referred to a purpose-built secure website (Vlaescu et al., 2016) with more information on the study, including the participation criteria. The participants were required to be Swedish citizens, at least 18 years of age, have access to the internet, and be fluent in Swedish. Information on the website also included the risks associated with participation, as well as the terms and conditions for participation. Volunteers were invited to submit their email addresses, and those who did were sent a link to complete an online screening questionnaire. The questionnaire included self-report measures of anxiety, depression, general well-being, and assertiveness, as well as questions regarding socio-demographics, experiences of psychological treatment, any current medication, and motivation for participation. Participants were not submitted to a clinical interview as part of the screening process. See Table 1 for a summary of the socio-demographic baseline characteristics.

**Table 1:** Socio-demographic baseline characteristics of participants.

Category		Waitlist	Unguided	Guided
	n	68	67	70
Age (years)	M	41	41	44
	SD	8	9	10
Sex (%)	Female	91	79	93
Civil status (%)	Single	37	36	29
	Partner	19	25	17
	Married	37	28	49
	Other	7	10	6
Highest education level (%)	Other	3	1	1
	Middle school	1	0	0
	High school/college	10	9	7
	Vocational training	7	4	7
	Currently at university	7	15	10
	University degree	71	70	74
Occupation (%)	Other	9	6	7
	Student	7	12	6
	Employed	74	76	76
	Unemployed	4	4	0
	Retired	0	0	4
	Parental leave	0	0	4
	Sick leave	6	1	3
Use of psychotropic medications (%)	No	76	70	67
	Yes, previously	7	9	17
	Yes, currently	16	21	16
Previous psychological treatment (%)	No	38	40	39
	Yes	62	60	61

In total, 657 individuals submitted their email addresses, of whom 464 completed the screening questionnaire. Of these, 126 were excluded for meeting the exclusion criteria, which were concurrent psychological treatment, a recent change in psychotropic medication, lack of time and/or motivation for participation, and a rating of 15 or above on the Patient Health Questionnaire (PHQ-9) measure of depression. The remaining 338 individuals were invited to participate in the study. Of these, 253 accepted the invitation.

### 2.3 Procedure

**Fig. 1:**
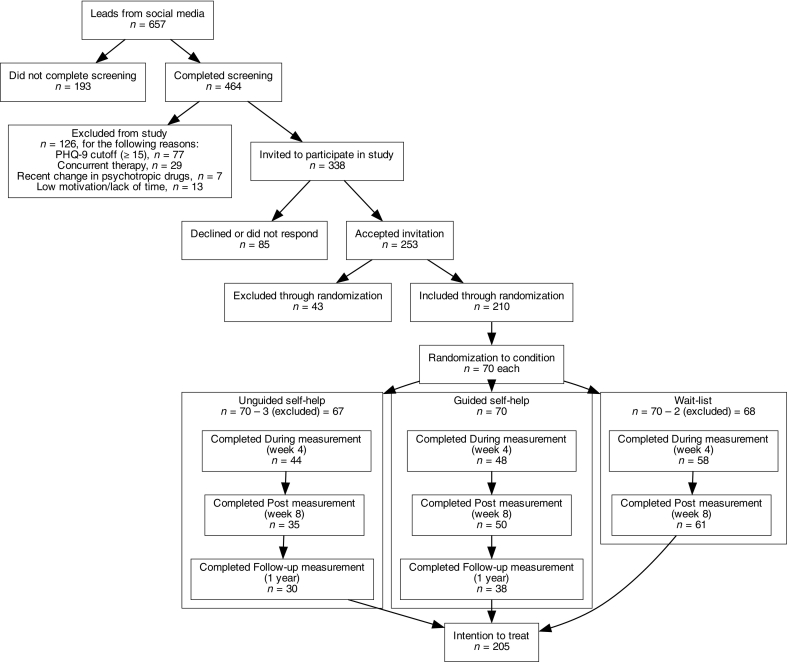
A total of 210 participants were included in the study through randomization and further randomized into three groups: unguided self-help, guided self-help, and wait-list, with 70 participants each.

Following the a priori power calculation, 210 participants were randomized to be included in the study. The remaining 43 individuals were offered access to the treatment materials but were excluded from all the analyses. The 210 participants were randomized to the three treatment conditions. The participants in the guided condition were randomized to one of two therapists. See Fig. 1 for a flow-chart detailing the recruitment and randomization steps. All randomization was performed by an independent third party at Stockholm University using random.org (Haahr, 2018) and sealedenvelope.com (Sealed Envelope Ltd., 2021).

### 2.4 Measures

The data were collected using the measures described below at four time points: week 0 (pre-treatment), week 4 (during the intervention), week 8 (post-treatment), and at a one-year follow-up. Participants in the wait-list condition were not included in the follow-up measurement since they had been given access to the unguided branch of the intervention after the post-treatment time point.

#### 2.4.1 Primary measures

Assertiveness style was measured using a Swedish translation (contributed by TH) of the Adaptive and Aggressive Assertiveness Scales (AAA-S; Thompson and Berenbaum, 2011), which contains 30 items, including “When someone I don't know well borrows something from me and forgets to return it, I… a. Demand it back; b. Ask if she/he is done and ask for it back” (a. and b. both scored from 1 = never to 5 = always). The English-language version of the AAA-S has good internal consistency for both aggressive assertiveness (.88) and adaptive assertiveness (.82). A Swedish translation (contributed by TH) of the Rathus Assertiveness Schedule (RAS; Rathus, 1973) was used as an additional measure of assertiveness style with 30 items, including “I find it embarrassing to return merchandise” (+3 = very characteristic of me, extremely descriptive to −3 = very uncharacteristic of me, extremely nondescriptive). The RAS has good internal consistency (.87) (Thompson and Berenbaum, 2011).

#### 2.4.2 Secondary measures

Depression was measured using the Patient Health Questionnaire 9-Item Scale (PHQ-9; Kroenke et al., 2010), which contains nine items, including “Feeling down, depressed, or hopeless” (0 = not at all to 3 = nearly every day). Generalized anxiety was measured using the Generalized Anxiety Disorder 7-item Scale (GAD-7; Spitzer et al., 2006), which contains seven items, including “Feeling nervous, anxious, or on edge” (0 = not at all to 3 = nearly every day). Social anxiety was measured using the Liebowitz Social Anxiety Scale (LSAS-SR; Fresco et al., 2001), which contains 24 items, including “Calling someone you don't know very well” (fear or anxiety, 0 = none to 3 = severe; avoidance, 0 = never to 3 = usually). General well-being was measured using the Questionnaire on well-being (Braconier, 2015), which contains 18 items, such as “To what extent during the past week have you felt calm and relaxed?” (0 = never to 5 = very often). These self-report measures have reported either good or excellent internal consistency (.89, .92, .96, and .93, respectively).

### 2.5 Intervention

The intervention was based on the Assert Yourself modules by Michel and Fursland (2008), which were adapted to Swedish by TH with permission from the copyright holders. The self-help material teaches the distinction between different types of assertiveness (constructive, aggressive, passive, and passive-aggressive). It also aids the reader in finding reasons to act more assertively and constructively. The material is inspired by and cites works by Alberti and Emmons (1974), Gambrill and Richey (1975), and Smith (1975), among others. In the material, assertiveness is described and operationalized based on Wolpe's (1990) theoretical assumptions regarding reciprocal inhibition and classic conditioning: By assertively practicing the expression of feelings, wishes, and demands in anxiety-evoking situations and relationships, where the person was previously prone to non-assertive behavior (e.g., subdued disappointment or anger), a person may experience less discomfort from autonomous anxiety responses over time. This is to be practiced in vivo, not just by acting. The long-term goal is to learn how to inhibit anxiety by being assertive. In cases where a physical counterpart is absent and anxiety is invoked by places, objects, or words, Wolpe (1952) suggests relaxation as a means to inhibit the anxiety response.

The material also includes a rationale for cognitive restructuring with methods by Beck (1979), Clark (1986), Clark and Wells (1995), and Powell (2017). Through behavioral experiments, readers test the validity of negative thoughts to achieve greater flexibility in their responses. Furthermore, the material includes a passage on progressive muscle relaxation for the reader to recognize bodily tension, reduce general strain, and practice an active coping technique for stressful situations. Chapters on specific challenges, such as saying “no,” dealing with criticism, and coping with disappointment, conclude the material.

The Swedish adaptation prompted additions to Michel and Fursland (2008), some of which are presented here. Based on recent research on exposure and inhibitory learning (e.g., Craske et al., 2008), the participants were encouraged to actively vary learning situations and work on new skills in as many environments as possible. In another new passage, inspired by the works on acceptance by Hayes (2004) and others, the participants were encouraged to actively search for and remain in the respondent discomfort they previously avoided. In line with Öst's (2006) recommendations for applied tension, progressive muscle relaxation was introduced early in the intervention and expanded over several weeks. The written material was also complemented by downloadable audio relaxation exercises, videos of conversations and role-playing, and several new interactive exercises.

Everything else being equal, the guided condition included time-limited (on average approximately 15 min per participant and week) asynchronous communication support from therapists having completed basic CBT training. Additional support for participants in the guided self-help condition included weekly messages on the treatment platform, involving feedback on homework, encouragement, validation, psychoeducation, and answers to any questions.

#### 2.5.1 Therapists

Both therapists working with participants in the guided condition were final-year clinical psychology students at Stockholm University. Both had completed basic training in CBT and received continuous supervision from a licensed psychotherapist with more than two decades of iCBT experience.

### 2.6 Data preparation

Five participants were excluded from the analyses due to wrongful inclusion, as they were receiving concurrent psychological treatment.

### 2.7 Analysis

All the data were analyzed using R 4.3.0, with the packages lmerTest (Kuznetsova et al., 2020), emmeans (Lenth, 2020), ggeffects (Lüdecke, 2018), performance (Lüdecke et al., 2023), and clinical significance (Claus, 2022). All the syntax is available at https://github.com/hmep/r2fu/, together with the anonymized data.

A linear mixed-effects model was fitted to estimate the fixed effects of group, time, and group-time interaction, and the random effects of participant (specifying a random intercept to control for individual differences), using an unstructured covariance pattern and the Restricted maximum likelihood (REML) estimation method. The Kenward-Roger approximations were used to estimate denominator degrees of freedom. Post-hoc pairwise comparisons of estimated marginal means were performed using *t*-tests. The significance of all the post-hoc tests was decided with Bonferroni-corrected *p*-values. The proportions of participants showing reliable change and reaching clinical significance were determined following Jacobson and Truax (1991), using the “c” definition to select the cutoff value. To honor the intention-to-treat principle in the analysis of clinical significance, the last observation was carried forward for any missing values. In a subsequent step, rank-sum tests of these proportions of clinically changed participants were performed to establish significance of differences between the waitlist and the treatment conditions.

## 3 Results

### 3.1 Treatment efficacy, primary, and secondary measures

**Fig. 2:**
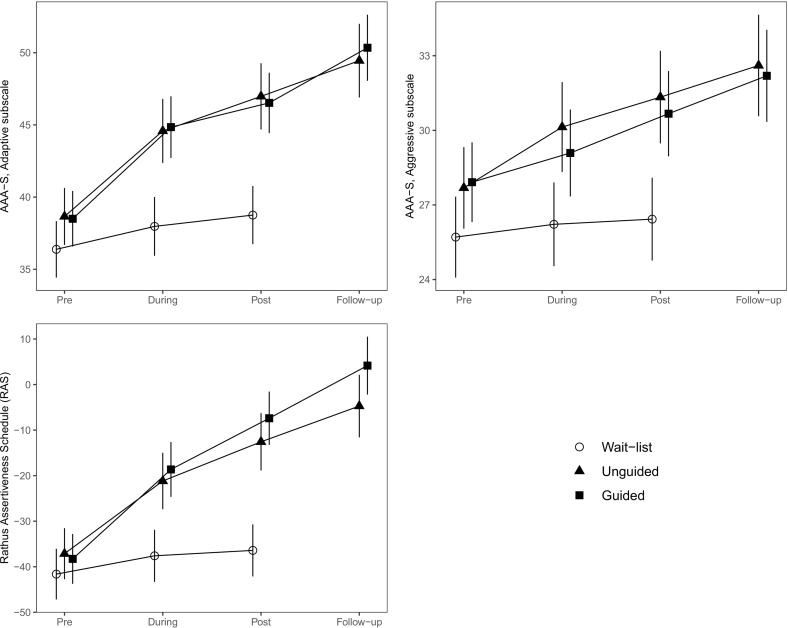
Plots of the estimated fixed effects for the primary transdiagnostic scales used to measure skillful, assertive behavior, and aggressive assertive behavior. The participants' estimated means for all three measures exhibited increasing levels of assertiveness during treatment in the unguided self-help and guided self-help groups, with negligible differences between the two treatment conditions.

**Fig. 3:**
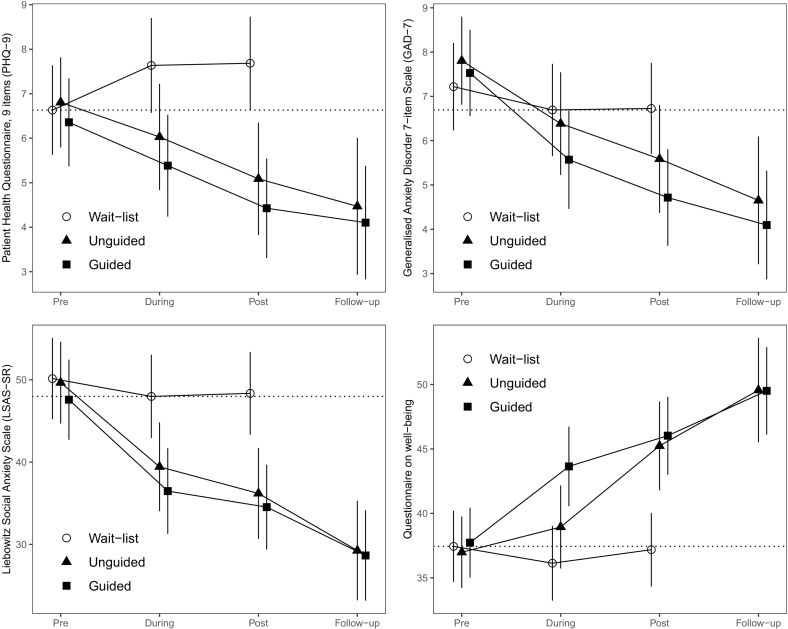
Plots of the estimated fixed effects for the secondary syndromal outcome measures, as well as for the general well-being measure. Participation in the unguided self-help and guided self-help conditions led to significant symptom alleviation between the pre and post time points as well as between the pre and one-year follow-up time points for depression and social anxiety. Participation in both treatment groups also led to significant increases in well-being. As with the transdiagnostic measures of assertive behavior, the differences between the post and follow-up time points were statistically inappreciable for all measures, in both treatment groups. The wait-list control group did not significantly change between any time points for either measure. However, to stay on the conservative side and counteract even the slightest nocebo effect of the wait-list condition, the most conservative estimate for the wait-list control condition was used in each follow-up between-group comparison; see the dotted line for a visual representation of the selected time point.

Mixed models that included the unguided self-help, guided self-help, and wait-list groups at the pre, during, and post time points revealed time–group interaction effects for all three measures of assertiveness: the AAA-S Adaptive subscale, F(4, 311.87) = 8.2, p < .001, the AAA-S Aggressive subscale, F(4, 308.68) = 2.95, p = .020, and the RAS, F(4, 316.44) = 19.54, p < .001; see Fig. 2. These interactions showed that the random assignment to group conditions had an effect over time on assertive behavior. Similarly, mixed models for the syndromal symptoms revealed time–group interaction effects for all four measures of anxiety, depression, and well-being: the PHQ-9, F(4, 320.11) = 4.55, p = .001, the GAD-7, F(4, 315.58) = 2.81, p = .026, the LSAS-SR, F(4, 302.49) = 8.72, p < .001, and the Well-being questionnaire, F(4, 313.62) = 7.5, p < .001; see Fig. 3. The estimated mean levels of depressive mood, generalized and social anxiety, and general well-being were significantly affected by participation in the intervention.

All subsequent post-hoc testing of marginal mean differences in pairwise comparisons included all groups and time points.

#### 3.1.1 Between-group effects on assertive behavior

Post-hoc testing revealed significant effects between both treatment groups and the wait-list group for all three primary measures of assertiveness at the post time point, that is, at the end of the eight-week Respekt^2^ treatment program. It also revealed significant effects at the follow-up one year after the end of treatment compared to the wait-list condition at the post time point. Table 2 summarizes the significance tests of the estimated marginal mean differences and effect sizes, with 95 % confidence intervals (CI).

**Table 2:** Within-group effect sizes [95 % CI] comparing estimated marginal means between Pre- and Post-treatment, as well as between Pre-treatment and 1-year Follow-up, and between group effect sizes [95 % CI] at post-treatment and 1-year Follow-up.

		Primary transdiagnostic measures of skillful behavior			Secondary measures of syndromal symptoms and well-being			
		AAA-S Adaptive	AAA-S Aggressive	RAS	PHQ-9	GAD-7	LSAS-SR	Well-being
Within-group effect sizes	Unguided, pre vs. post	1.01 [0.76, 1.26]⁎⁎⁎	−0.53 [−0.75, −0.31]⁎⁎⁎	1.05 [0.82, 1.28]⁎⁎⁎	0.41 [0.10, 0.71]	0.53 [0.25, 0.82]⁎	0.65 [0.45, 0.84]⁎⁎⁎	0.71 [0.42, 1.00]⁎⁎⁎
	Unguided, pre vs. follow-up	1.31 [1.02, 1.60]⁎⁎⁎	−0.72 [−0.97, −0.47]⁎⁎⁎	1.39 [1.12, 1.65]⁎⁎⁎	0.55 [0.18, 0.92]	0.76 [0.42, 1.10]⁎⁎⁎	0.98 [0.75, 1.21]⁎⁎⁎	1.09 [0.74, 1.43]⁎⁎⁎
	Unguided, post vs. follow-up	0.30 [0.00, 0.60]	−0.19 [−0.45, 0.08]	0.34 [0.07, 0.60]	0.15 [−0.25, 0.54]	0.23 [−0.14, 0.59]	0.33 [0.10, 0.57]	0.37 [0.01, 0.74]
	Guided, pre vs. post	0.98 [0.75, 1.20]⁎⁎⁎	−0.40 [−0.60, −0.21]⁎⁎	1.32 [1.11, 1.53]⁎⁎⁎	0.46 [0.18, 0.73]	0.68 [0.42, 0.93]⁎⁎⁎	0.63 [0.46, 0.80]⁎⁎⁎	0.72 [0.46, 0.97]⁎⁎⁎
	Guided, pre vs. follow-up	1.44 [1.18, 1.70]⁎⁎⁎	−0.63 [−0.85, −0.40]⁎⁎⁎	1.81 [1.57, 2.05]⁎⁎⁎	0.53 [0.23, 0.84]⁎	0.83 [0.53, 1.12]⁎⁎⁎	0.91 [0.71, 1.11]⁎⁎⁎	1.02 [0.73, 1.31]⁎⁎⁎
	Guided, post vs. follow-up	0.46 [0.21, 0.72]⁎	−0.22 [−0.45, 0.00]	0.49 [0.26, 0.73]⁎⁎	0.08 [−0.24, 0.40]	0.15 [−0.15, 0.45]	0.28 [0.09, 0.48]	0.30 [0.01, 0.59]
	Wait-list, pre vs. post	0.29 [0.09, 0.49]	−0.11 [−0.29, 0.07]	0.22 [0.03, 0.41]	−0.25 [−0.50, 0.01]	0.12 [−0.12, 0.36]	0.09 [−0.07, 0.24]	−0.02 [−0.25, 0.21]
Between-group effect sizes	Unguided at post vs. wait-list at post	1.00 [0.62, 1.38]⁎⁎⁎	−0.72 [−1.09, −0.35]⁎⁎	1.02 [0.65, 1.39]⁎⁎⁎	0.61 [0.22, 1.01]	0.27 [−0.11, 0.66]	0.59 [0.22, 0.95]	0.70 [0.31, 1.08]⁎
	Unguided at follow-up vs. wait-list at post	1.30 [0.90, 1.71]⁎⁎⁎	−0.90 [−1.29, −0.51]⁎⁎⁎	1.35 [0.96, 1.75]⁎⁎⁎	0.76 [0.32, 1.20]⁎	0.50 [0.07, 0.93]	0.92 [0.54, 1.30]⁎⁎⁎	1.07 [0.63, 1.50]⁎⁎⁎
	Unguided at follow-up vs. wait-list at^a^	(idem)	(idem)	(idem)	0.51 [0.07, 0.95]	0.49 [0.06, 0.92]	0.90 [0.52, 1.29]⁎⁎⁎	1.05 [0.62, 1.47]⁎⁎⁎
	Guided at post vs. wait-list at post	0.95 [0.59, 1.30]⁎⁎⁎	−0.62 [−0.97, −0.27]⁎	1.24 [0.88, 1.60]⁎⁎⁎	0.77 [0.40, 1.14]⁎⁎	0.48 [0.12, 0.85]	0.67 [0.32, 1.02]⁎	0.76 [0.40, 1.13]⁎⁎
	Guided at follow-up vs. wait-list at post	1.41 [1.03, 1.79]⁎⁎⁎	−0.84 [−1.21, −0.47]⁎⁎⁎	1.73 [1.36, 2.11]⁎⁎⁎	0.85 [0.45, 1.24]⁎⁎	0.63 [0.25, 1.02]	0.95 [0.58, 1.31]⁎⁎⁎	1.06 [0.67, 1.45]⁎⁎⁎
	Guided at follow-up vs. wait-list at^a^	(idem)	(idem)	(idem)	0.60 [0.21, 0.98]	0.63 [0.24, 1.02]	0.93 [0.57, 1.30]⁎⁎⁎	1.04 [0.66, 1.43]⁎⁎⁎
	Guided vs. unguided at post	−0.05 [−0.43, 0.32]	0.10 [−0.27, 0.47]	0.22 [−0.15, 0.59]	0.16 [−0.24, 0.56]	0.21 [−0.18, 0.60]	0.08 [−0.29, 0.45]	0.07 [−0.33, 0.47]
	Guided vs. unguided at follow-up	0.11 [−0.31, 0.53]	0.06 [−0.34, 0.46]	0.38 [−0.02, 0.78]	0.09 [−0.39, 0.56]	0.13 [−0.32, 0.59]	0.03 [−0.37, 0.42]	−0.00 [−0.46, 0.45]

CI = confidence interval.

Pre = pre-treatment measurement at 0 weeks; During = measurement during week 4; Post = measurement after completion of treatment at week 8; Follow-up = measurement at 1 year after completion.

a The most conservative measurement for the Wait-list control condition, in order to suppress any nocebo effects; see dotted lines in graphs in Fig. 3 for identification of time point. p-Values are Bonferroni adjusted, based on pairwise comparisons of all sampled time points and conditions.

⁎ p < .05.

⁎⁎ p < .01.

⁎⁎⁎ p < .00;

Compared to the wait-list condition, the effect of the unguided self-help condition on adaptive assertiveness using the AAA-S Adaptive subscale primary measure was large at the post time point, *∆M* = 8.2, *t*(381) = −5.29, *p*_*Bonf*_ < .001, *ES* = 1.00, and even larger at the follow-up, *∆M* = 10.7, *t*(439) = −6.45, *p*_*Bonf*_ < .001, *ES* = 1.30. Large negative effects at the post time point were found for aggressive assertiveness as measured with the AAA-S Aggressive subscale, *∆M* = −4.9, *t*(345) = −3.85, *p*_*Bonf*_ = .008, *ES* = −0.72, and at the follow-up, *∆M* = −6.2, *t*(397) = −4.60, *p*_*Bonf*_ < .001, *ES* = −0.90. Large effects were also found for “compound” assertiveness assessed with the RAS, *∆M* = 23.8, *t*(353) = −5.50, *p*_*Bonf*_ < .001, ES = 1.02, and at the follow-up, *∆M* = 31.7, *t*(402) = −6.96, *p*_*Bonf*_ < .001, *ES* = 1.35.

Similarly, the effect of the guided self-help condition on the AAA-S Adaptive subscale was large at the post time point, *∆M* = 7.8, *t*(346) = −5.25, *p*_*Bonf*_ < .001, *ES* = 0.95, reaching *∆M* = 11.6, *t*(393) = −7.46, *p*_*Bonf*_ < .001, *ES* = 1.41 at the follow-up. Large negative effects at the post time point were identified for the AAA-S Aggressive subscale, *∆M* = −4.2, *t*(315) = −3.48, *p*_*Bonf*_ = .032, *ES* = −0.62, and at the follow-up, *∆M* = −5.8, *t*(355) = −4.54, *p*_*Bonf*_ < .001, *ES* = −0.84. Large effects were also found for the RAS, *∆M* = 29.0, *t*(327) = −6.96, *p*_*Bonf*_ < .001, *ES* = 1.24 at the post time point, and *∆M* = 40.6, *t*(371) = −9.31, *p*_*Bonf*_ < .001, *ES* = 1.73 at the follow-up.

Comparing the unguided self-help and guided self-help conditions, no significant differences were found either at the post or the follow-up time points, revealing that the participants working through the intervention on their own fared as well as those who were supported by a therapist.

#### 3.1.2 Between-group effects on syndromal symptoms of anxiety, depression, and well-being

In the post-hoc testing, no effect on depressive symptoms, measured with the PHQ-9, was found at the post time point comparing the wait-list and the unguided self-help groups. Tentative evidence was found for therapist support benefiting depressed participants: The wait-list versus the guided self-help comparison revealed a moderate to large effect on PHQ-9 at the post time point, *∆M* = 3.3, *t*(435) = 4.16, *p*_*Bonf*_ = .002, *ES* = 0.77. However, this effect dissipated when the follow-up comparison was instead made against the most conservative value collected in the wait-list group, in this case from the pre time point; see Figure for a visual exploration of a possible nocebo effect.

No between-group effects were found for the GAD-7 when comparing the treatment and the wait-list groups. In addition, there was no significant effect of the unguided self-help condition on social anxiety symptoms measured with the LSAS-SR at the post time point. However, a large effect was found at the follow-up, *∆M* = 18.7, *t*(364) = 4.66, *p*_*Bonf*_ < .001, *ES* = 0.90 for the unguided self-help group, compared with the lowest value collected at the during time point. For the guided self-help group, a moderate effect was found at the post time point, *∆M* = 13.8, *t*(286) = 3.76, *p*_*Bonf*_ = .011, *ES* = 0.67, with a large effect at the follow-up, *∆M* = 19.3, *t*(321) = 5.07, *p*_*Bonf*_ < .001, *ES* = 0.93, this time compared with the most conservative value, from the during time point, for the wait-list group.

For the Well-being Questionnaire, moderate effects were found at post in both the unguided and the guided groups, *∆M* = 8.1, *t*(443) = −3.53, *p*_*Bonf*_ = .026, *ES* = 0.70, and *∆M* = 8.8, *t*(396) = −4.17, *p*_*Bonf*_ = .002, *ES* = 0.76, respectively, increasing to large effects at follow-up in both treatment groups, *∆M* = 12.1, *t*(504) = −4.85, *p*_*Bonf*_ < .001, *ES* = 1.05, and *∆M* = 12.1, *t*(440) = −5.41, *p*_*Bonf*_ < .001, *ES* = 1.04, respectively.

Thus, the eight-week Respekt^2^ intervention did not affect either depression or generalized anxiety. However, it did have a pronounced effect on social anxiety, and on general well-being. See Table 2 for a summary of the significant effects, including 95 % CIs.

#### 3.1.3 Within-group effects

As shown in Table 2, the participants in both treatment groups enjoyed sustained within-group effects on assertiveness at the follow-up compared to the pre-treatment time point, measured with the AAA-S Adaptive and Aggressive subscales and the RAS. Thus, assertive behavior was still manifested well beyond the end of participation in the intervention. However, significant effects between the post and the follow-up time points were found only for two measures in the guided self-help group, where the AAA-S Adaptive subscale and the RAS exhibited small to moderate effects, *∆M* = 3.8, *t*(369) = −3.64, *p*_*Bonf*_ = .017, *ES* = 0.46 and *∆M* = 11.6, *t*(380) = −4.20, *p*_*Bonf*_ = .002, *ES* = 0.49, respectively. In the unguided self-help group, no difference between the post and the follow-up time points was found for either measure, *p*_*Bonf*_ = 1 and *p*_*Bonf*_ = .731, indicating that the therapist support provided some benefit to the participants' ability to generalize adaptive assertive behaviors beyond the duration of the intervention.

Among the secondary measures of syndromal symptoms, depression, as captured with the PHQ-9, decreased significantly from the pre time point only in the guided self-help group and only at the follow-up, showing a medium effect, *∆M* = 2.3, *t*(394) = 3.42, *p*_*Bonf*_ = .038, *ES* = 0.53, implying that therapist support benefited depressed participants on a longer rather than a shorter time scale.

### 3.2 Clinical significant change

#### 3.2.1 Reliable recovery with regard to assertive behavior

Adding to the picture that assertive behavior increased as a consequence of participation in the intervention, a significant difference between the groups at the post time point was found in the proportion of participants who had recovered clinically (i.e., had moved across the cutoff for reliable and clinically significant change), with respect to the AAA-S Adaptive subscale, *χ*^*2*^(2) = 7.92, *p* = .019, and the RAS, *χ*^*2*^(2) = 14.92, *p* < .001. At the follow-up, the proportions of the recovered category were also significantly different for the AAA-S Adaptive subscale, *χ*^*2*^(2) = 12.35, p = .002 and for the RAS, *χ*^*2*^(2) = 21.51, *p* < .001. However, no differences in the proportions of recovered participants with regard to the AAA-S Aggressive subscale were found at either time point.

Notably, 13 participants (19%) in the unguided self-help group experienced clinical recovery with regard to adaptive assertiveness measured with the AAA-S Adaptive subscale at the post time point, increasing to 15 participants (22%) at the follow-up. In the guided self-help group, the number of participants recovered at the post time point was not significantly different from that of the wait-list group, while it was significantly larger at the follow-up, with 18 participants (26%). For assertiveness assessed with the RAS, the corresponding numbers (and percentages) were 17 participants (25%) for the unguided self-help condition and 20 participants (29%) for the guided self-help condition at the post time point, increasing to 21 participants (31%) and 25 participants (36%), respectively, at the follow-up.

Thus, the clinical significance findings are mostly in agreement with the statistical analysis of the change, confirming that adaptive expressions of assertiveness in both treatment groups increased from the pre to the post time points and beyond, while deviating with regard to aggressive expressions.

#### 3.2.2 Reliable recovery with regard to syndromal symptoms

As for the syndromal symptoms, the proportions of those with reliable recovery from social phobia, as captured with the LSAS-SR, were significantly different between the groups at the post time point, *χ*^*2*^(2) = 7.54, *p* = .023. However, no difference between the groups was found at the post time point with regard to the PHQ-9 or GAD-7. At the follow-up, the difference in the proportions of recovered participants between the conditions was significant for the PHQ-9, *χ*^*2*^(2) = 11.87, *p* = .003, and the LSAS-SR, *χ*^*2*^(2) = 14.2, *p* < .001, but not for the GAD-7.

Clinical recovery measured with the PHQ-9 between the pre and post time points indicated that the intervention compared to the wait-list condition was effective in taking 16 participants (23%) out of depression in the guided self-help group, but none in the unguided self-help group, suggesting that interaction with a therapist may aid recovery in depressed participants. In addition, a significant number of participants in the unguided self-help group recovered from social anxiety, as captured with the LSAS-SR, 11 participants (16%), at the post time point, increasing to 14 participants (21%) at the follow-up. In the guided self-help condition, no significant number of participants was found to have recovered from social anxiety at the post time point, however, 18 participants (26%) were found to have recovered at the follow-up. In other words, although there was not immediate effect on social anxiety, symptoms did subside in the year following treatment to the point that a meaningful number of participants had recovered. Combined with the clinically relevant recovery to functional levels of assertive behavior measured with the AAA-S Adaptive subscale and the RAS, this could be a sign of generalization of assertive behavior having continued after the end of treatment, facilitating extinction of autonomous anxiety responses and/or reducing avoidance and escape behaviors from them.

As for general well-being, 18 participants (26%) moved into the recovered category in the guided self-help group, but none in the unguided group, again indicating a possible benefit from therapist support.

Table 3 provides a summary of the numbers and proportions of clinical recovery in the different groups and the significance, if any, of the pairwise tests of proportions against those of the wait-list group.

**Table 3:** Clinical significance summary of the number (and proportion in %) of participants that changed reliably and moved from the clinical to the functional population from Pre-treatment to Post- and 1-year Follow-up-time points respectively (rows named ‘Recovered’). For missing values (i.e., caused by drop-outs), the last collected value was moved forward to the next measurement time point, in order to respect the intention to treat principle.

		Wait-list	Unguided self-help		Guided self-help	
		Pre–post	Pre–post	Pre–follow-up	Pre–post	Pre–follow-up
AAA-S adaptive	Recovered	3 (4 %)	13 (19 %)⁎	15 (22 %)⁎	13 (19 %)	18 (26 %)⁎⁎
	Improved	8 (12 %)	4 (6 %)	6 (9 %)	6 (9 %)	5 (7 %)
	Unchanged	57 (84 %)	50 (75 %)	46 (69 %)	51 (73 %)	47 (67 %)
	Deteriorated	0 (0 %)	0 (0 %)	0 (0 %)	0 (0 %)	0 (0 %)
	Harmed	0 (0 %)	0 (0 %)	0 (0 %)	0 (0 %)	0 (0 %)
AAA-S aggressive	Recovered	3 (4 %)	0 (0 %)	0 (0 %)	0 (0 %)	0 (0 %)
	Improved	1 (1 %)	1 (1 %)	0 (0 %)	1 (1 %)	1 (1 %)
	Unchanged	55 (81 %)	56 (84 %)	55 (82 %)	61 (87 %)	55 (79 %)
	Deteriorated	3 (4 %)	6 (9 %)	7 (10 %)	4 (6 %)	5 (7 %)
	Harmed	6 (9 %)	4 (6 %)	5 (7 %)	4 (6 %)	9 (13 %)
RAS	Recovered	3 (4 %)	17 (25 %)⁎⁎	21 (31 %)⁎⁎⁎	20 (29 %)⁎⁎	25 (36 %)⁎⁎⁎
	Improved	3 (4 %)	11 (16 %)	9 (13 %)	13 (19 %)	13 (19 %)
	Unchanged	62 (91 %)	39 (58 %)	37 (55 %)	37 (53 %)	32 (46 %)
	Deteriorated	0 (0 %)	0 (0 %)	0 (0 %)	0 (0 %)	0 (0 %)
	Harmed	0 (0 %)	0 (0 %)	0 (0 %)	0 (0 %)	0 (0 %)
PHQ-9	Recovered	3 (4 %)	10 (15 %)	11 (16 %)	16 (23 %)⁎⁎	18 (26 %)⁎⁎
	Improved	4 (6 %)	5 (7 %)	6 (9 %)	4 (6 %)	5 (7 %)
	Unchanged	49 (72 %)	47 (70 %)	48 (72 %)	45 (64 %)	40 (57 %)
	Deteriorated	8 (12 %)	1 (1 %)	0 (0 %)	3 (4 %)	2 (3 %)
	Harmed	4 (6 %)	4 (6 %)	2 (3 %)	2 (3 %)	5 (7 %)
GAD-7	Recovered	8 (12 %)	13 (19 %)	14 (21 %)	14 (20 %)	19 (27 %)
	Improved	5 (7 %)	7 (10 %)	9 (13 %)	4 (6 %)	4 (6 %)
	Unchanged	47 (69 %)	42 (63 %)	38 (57 %)	51 (73 %)	44 (63 %)
	Deteriorated	6 (9 %)	2 (3 %)	2 (3 %)	1 (1 %)	2 (3 %)
	Harmed	2 (3 %)	3 (4 %)	4 (6 %)	0 (0 %)	1 (1 %)
LSAS-SR	Recovered	2 (3 %)	11 (16 %)⁎	14 (21 %)⁎	11 (16 %)	18 (26 %)⁎⁎
	Improved	7 (10 %)	13 (19 %)	12 (18 %)	12 (17 %)	10 (14 %)
	Unchanged	52 (76 %)	41 (61 %)	40 (60 %)	47 (67 %)	41 (59 %)
	Deteriorated	5 (7 %)	1 (1 %)	1 (1 %)	0 (0 %)	0 (0 %)
	Harmed	2 (3 %)	1 (1 %)	0 (0 %)	0 (0 %)	1 (1 %)
Well-being	Recovered	6 (9 %)	11 (16 %)	13 (19 %)	14 (20 %)	18 (26 %)⁎
	Improved	0 (0 %)	5 (7 %)	6 (9 %)	4 (6 %)	7 (10 %)
	Unchanged	56 (82 %)	47 (70 %)	46 (69 %)	51 (73 %)	44 (63 %)
	Deteriorated	2 (3 %)	1 (1 %)	0 (0 %)	0 (0 %)	1 (1 %)
	Harmed	4 (6 %)	3 (4 %)	2 (3 %)	1 (1 %)	0 (0 %)

AAA-S Adaptive = Adaptive and Aggressive Assertiveness Scales, Adaptive subscale; AAA-S Aggressive = Adaptive and Aggressive Assertiveness Scales, Aggressive Subscale, RAS = Rathus Assertiveness Schedule; PHQ-9 = Patient Health Questionnaire, 9 items; GAD-7 = Generalized Anxiety Disorder 7-item Scale; LSAS-SR = Liebowitz Social Anxiety Scale; Well-being = Questionnaire on Well-being.

p-values are Bonferroni adjusted, based on rank-sum tests of the proportions of clinically improved participants between the three conditions at the post and follow-up time points, respectively.

⁎ p < .05.

⁎⁎ p < .01.

⁎⁎⁎ p < .00

#### 3.2.3 Reliable deterioration and harm

Checking for any reliable signs of harm in the most severe of the adverse outcome categories (see Table 3), significant differences between the groups in clinically significant change were found for the Well-being scale. Post-hoc testing revealed that the number of participants having fallen into the “harmed” category (6 participants; 9%) in the wait-list group, was significantly larger than in the guided group at both the post time-point and the follow-up time-point. No other instances of suspected reliable harm between the pre and the post time points or the pre to the follow-up time points were identified for either measure.

The second to worst category in assessing reliable change is “deteriorated.” To identify any possible cases of reliable deterioration and reliable harm, these two categories were collapsed into the conservative ad hoc category “worsened” gathering participants who had moved into either category. Applying the “worsened” portmanteau category, a significant difference between the groups for the LSAS-SR was found, meriting a follow-up pairwise comparison. This revealed that the number of participants (7 participants; 10%) in the wait-list condition who had reached either “deteriorated” or “harmed” between pre and post treatment, was significantly larger than the corresponding number in the guided self-help group. The same held true when comparing the pre to the follow-up time points, where these individuals were significantly more than the number of worsened participants in both the unguided and the guided self-help groups. Finally, a difference between the groups was found for the PHQ-9, with the post-hoc testing revealing a difference for the guided self-help group, with 12 worsened participants (18%).

In summary, these findings reveal that the non-active wait-list condition brought about adverse clinical change for 9% of the participants with regard to general well-being, 10% with regard to social anxiety, and 18% with regard to depression.

## 4 Discussion

This randomized controlled trial tested the effects of a transdiagnostic internet intervention targeting assertiveness (Respekt^2^). The findings indicate that the treatment led to increased assertive behavior and reduced psychiatric symptoms. Having been largely ignored as a construct in clinical psychology research since the 1990s, our study provides new data on assertiveness as a viable transdiagnostic stand-alone dependent variable in psychological treatment.

The large effects on assertiveness measured with the AAA-S Adaptive subscale are comparable to those found in clinical trials of iCBT interventions for other transdiagnostic behavioral problems. Measurement with the RAS at the follow-up confirms these effects, as does the insignificantly larger effects found for assertiveness measured with the AAA-S Adaptive subscale and the RAS in the guided self-help group. Benchmarking against procrastination (Rozental et al., 2015) and perfectionism (Rozental et al., 2017), these results indicate that the assertiveness construct can be used successfully as a behavior therapy target with various clinical presentations, helping participants to appreciate and report changes in healthy assertion levels in their daily lives.

It should be noted that while there were significant negative effects on aggressive assertiveness in both treatment groups, no statistically significant number of clinically worsened participants was found at either the post or the follow-up time points for the AAA-S Aggressive subscale in either group, compared to the wait-list. This should not be surprising, however, since for this particular sample the baseline levels of aggressive assertiveness were very low. Also, the operationalization of aggressive assertiveness is fuzzy and prone to individual differences in interpretation; behavior that one person deems aggressive assertion might be healthy assertion to another. Likewise, what counts as healthy assertion in one specific societal/cultural context might be perceived as normatively aggressive in another (Mitamura, 2018). Therefore, for a particular individual in a particular context, it is probably warranted to track only how the relationship between levels of adaptive and aggressive assertiveness changes over time, taking into account that individual's idiographic goals in therapy. This topic may well be further explored in future studies. For the purposes of this study, however, the lack of clinical levels of aggressive assertiveness indicates that participation did not lead to possibly adverse effects in relationships.

The effects at the follow-up on social anxiety and depression are in agreement with those for iCBT in general (Andrews et al., 2018), which in turn is about the same as for face-to-face treatment (Hedman-Lagerlöf et al., 2023; Carlbring et al., 2018), with the effects on general well-being of rounding out the picture of benefits from participation. However, the current intervention was insufficient for ameliorating generalized anxiety. Possibly, the overall structure of the Respekt^2^ intervention, with its emphasis on cheerleading participants in designing and performing in vivo behavioral experiments early on in treatment and for a limited time, was not adequate for addressing generalized anxiety symptoms where non-commitment to exposure and behavioral rigidity are often important first hurdles to overcome.

The analysis of clinical change revealed that large majorities in all groups at both the post and follow-up time points remained unchanged. This is expected for a non-clinical sample, for which baseline measurement scores were already mostly well on the functional side of cutoff values.

### 4.1 Limitations and future directions

The guided and unguided conditions yielded similar average levels across assertiveness measures as well as indicators of psychological problem symptoms, potentially indicating a ceiling effect of the self-help material when applied to a non-clinical population. Future studies should explore the impact of therapist involvement (possibly AI-enhanced, Carlbring et al., 2023) and its interplay with participant factors in greater detail for diverse diagnostic populations. Ideally, these studies would involve a larger number of therapists and incorporate therapist factors into the analysis (Magnusson et al., 2018). This approach could reveal how varying levels of therapist support (ranging from positive reinforcement to troubleshooting, Berg et al., 2022) influence retention rates, engagement, and ultimately, the outcomes and cost-benefit ratios for different diagnostic subgroups.

The study has a number of additional limitations that impair the generalizability of the findings. In studies of iCBT, the recruitment method is one of the most important factors influencing the symptom burden of the sample under investigation (Lindner et al., 2015). Recruitment for this study was performed via advertising on social media, where the presentation of the ads by revenue-maximizing design was skewed to boost click-throughs by the algorithms employed by the respective ad networks. We achieved distributions with regard to sex (79–91% female participants), higher education level (70–74%) and previous participation in therapy (60–62%) that are higher than expected had the sampling been purely random. Generalization of the findings needs to be made with caution before being confirmed with other samples in future studies.

Another methodological drawback of the current study is that some of the measurement scales were recently translated into Swedish without back translation, somewhat impairing the ability to compare findings with the data for the English-speaking populations where the scales were originally validated. The Swedish adaptations of the RAS and AAA-S scales should be quality controlled with back translation prior to future usage and, if possible, also validated for Swedish clinical and non-clinical populations. For the assessment of general well-being, a more established measure than the Well-being Questionnaire should preferably be used.

It is also noteworthy that the mean levels of depression in the wait-list control group increased as the participants waited in line to begin treatment; this might be due to a nocebo or reverse placebo effect, where participants' expectations contribute to their mood worsening (Furukawa et al., 2014), which in turn risks inflating between-group effect sizes. Analysis of reliable change confirmed this hypothesis, revealing that 12 individuals (18%) in the wait-list group worsened while waiting for treatment. To cancel out this nocebo effect in the analysis, the most conservative estimated marginal mean from either of the pre, during, or post time points was used for the follow-up comparison (in effect, underestimating rather than overestimating the difference). In any future studies, researchers would be wise to employ an active wait-list condition, such as participation in a discussion forum, to avoid running the risk of artificially inflated effect sizes (Cuijpers et al., 2016).

Preferably, any future replication or extended version of the current study should also collect data at no less than four time points, allowing data to be fitted not only using random intercepts (controlling for/capturing initial differences between subjects) but also with random slopes (controlling for/capturing individual trajectories). As noted above, it might also be necessary to add a third level to the model to control for therapist factors.

Along with the third wave of CBT, transdiagnostic behavioral approach goals have gained ground; that is, those captured with the Valued Living Questionnaire (VLQ; Wilson et al., 2010), the Acceptance and Action Questionnaire (AAQ-II; Fledderus et al., 2012; Lundgren and Parling, 2017), or, more generally, the Process-Based Assessment Tool (PBAT; Ciarrochi et al., 2022). Further exploration of how the pursuit of healthy assertion goals might influence these and other similar constructs could illuminate what goals are best suited for different patients (or populations) to find acceptable targets that can help short-circuit verbally expressed defenses head-on and thus increase the likelihood of engagement in new learning in CBT. Rhetorically, who does not want to be better still at respectfully asserting their feelings, wishes, and needs?

In the current study, we found that participation in Respekt^2^ increased assertive expressions, and at the same time reduced self-assessed social anxiety in a non-clinical sample. The intervention did not have an immediate effect on generalized anxiety, although there was some within-group evidence of beneficial longer-term effects on depression. With only a few exceptions, the guided condition did not yield better outcomes than the unguided condition, lending support for Respekt^2^ as a stand-alone, self-help intervention. Overall, the findings demonstrate that assertiveness is a potentially useful target in CBT and iCBT in the treatment of both psychiatric syndromes and non-syndromal problems in daily life, calling for more research on the construct in various applications.

## Declaration of competing interest

No financial conflicts exist. Author Michel, creator of the "Assert Yourself" modules used in this study, gains no royalties from their free public accessibility (Department of Health, Western Australia.).
